# Association of the European Society for Medical Oncology Magnitude of Clinical Benefit Scale (ESMO-MCBS) Scores with Guideline Recommendations in Breast Cancer

**DOI:** 10.3390/curroncol33040227

**Published:** 2026-04-17

**Authors:** Bushra Salman, Intissar Yehia, Hadil Al Sharqi, Roula Al Shidi, Miaad A. Al Dhahri, Saba Al Ghefeili, Meriem Makhloufi, Adil Al Ajmi, Suhaila Al Farsi, Zayana Al Kiyumi, Zaid Riyadh Raouf Al Ishaq, Omar Abdelhakim Ayaad, Khalid Al Baimani

**Affiliations:** 1 Pharmacy Department, National Hematology and Bone Marrow Transplant Center, University Medical City, Muscat 123, Oman; b.salman@squ.edu.om; 2 Medical Oncology Department, Sultan Qaboos Comprehensive Cancer Care and Research Center, University Medical City, Muscat 123, Oman; i.yehia@cccrc.gov.om (I.Y.); m.khadraoui@cccrc.gov.om (M.M.); 3 Department of Pharmacy, Sultan Qaboos Comprehensive Cancer Care and Research Center, University Medical City, Muscat 123, Oman; h.alsharqi@cccrc.gov.om (H.A.S.); r.alshidi@cccrc.gov.om (R.A.S.); s.alghefeili@cccrc.gov.om (S.A.G.); 4 Sultan Qaboos Comprehensive Cancer Care and Research Center, University Medical City, Muscat 123, Oman; m.aldhahri@cccrc.gov.om (M.A.A.D.); s.alfarsi-1@cccrc.gov.om (S.A.F.); z.alkiyumi@cccrc.gov.om (Z.A.K.); 5 Department of Surgery, Sultan Qaboos Comprehensive Cancer Care and Research Center, University Medical City, Muscat 123, Oman; a.aljarrah@cccrc.gov.om (A.A.A.); z.alishaq@cccrc.gov.om (Z.R.R.A.I.); 6 Department of Quality and Accreditation, Sultan Qaboos Comprehensive Cancer Care and Research Center, University Medical City, Muscat 123, Oman; o.ayaad@cccrc.gov.om

**Keywords:** breast cancer, ESMO Magnitude of Clinical Benefit Scale (ESMO-MCBS), clinical practice guidelines, guideline concordance, value-based oncology, systemic therapy, metastatic breast cancer, evidence-based medicine

## Abstract

Objectives: To evaluate the association between European Society for Medical Oncology Magnitude of Clinical Benefit Scale (ESMO-MCBS) scores and treatment recommendations from European Society for Medical Oncology, National Comprehensive Cancer Network (NCCN), and American Society of Clinical Oncology (ASCO) in curative and metastatic breast cancer (BC), and to assess inter-guideline concordance. Methods: We conducted a comparative review of 47 systemic BC therapies with published ESMO-MCBS scores (accessed 30 June 2025). Guideline recommendations from ESMO, NCCN, and ASCO were extracted from the most recent versions and harmonized into ordinal tiers. Associations between MCBS scores and recommendation categories were evaluated using Fisher’s exact test and Spearman’s rank correlation in the palliative setting. Curative therapies were analyzed descriptively due to limited variability. Results: Among 38 palliative therapies, 40% achieved high clinical benefit (MCBS 4–5). A significant association was observed between MCBS scores and NCCN recommendations (*p* = 0.003; ρ = 0.48), with 62% of NCCN Category 1 therapies demonstrating high benefit versus 13% in other categories. No significant associations were observed with ASCO (*p* = 0.101; ρ = 0.18) or ESMO guideline recommendations (*p* = 0.073; ρ = 0.19). Inter-guideline concordance was strongest between ASCO and ESMO (*p* = 0.033; ρ = 0.48). In the curative setting (*n* = 9), most therapies received an MCBS grade A and top-tier guideline endorsement. Conclusions: Alignment between ESMO-MCBS and guideline recommendations is moderate and framework-dependent, strongest with NCCN in metastatic BC. Discordance primarily reflects differences between magnitude-of-benefit thresholds and evidence-certainty frameworks. ESMO-MCBS may serve as a complementary tool to enhance value-based clinical and policy decision-making.

## Simple Summary

Cancer treatment guidelines are widely used to support clinical decision-making, but they differ in how they evaluate and recommend therapies. Some systems focus on the strength and certainty of evidence, while others aim to quantify how much benefit a treatment provides to patients, for example, in terms of survival duration or quality of life. In this study, we compared these approaches in breast cancer by examining how treatment recommendations align with the magnitude of clinical benefit and with each other. We found that agreement between systems is only partial, particularly for treatments with modest benefits, reflecting differences in how value is defined. Importantly, tools that measure the size of benefit can complement, rather than replace, guideline recommendations by providing additional context for decision-making. These findings may help clinicians, policymakers, and healthcare systems better interpret treatment recommendations, prioritize therapies, and support more transparent and value-based care.

## 1. Background

The treatment landscape in breast cancer (BC) has undergone a tremendous evolution in recent decades, with major improvements in overall survival (OS) and health-related quality of life (QoL). In many developed countries, five-year survival rates now exceed 90% due to the availability of early detection strategies, multimodal care, and the use of novel systemic therapies [1]. Concurrent with these advances, the rapid expansion of therapeutic options has increased the complexity of clinical decision-making, especially in the presence of marginal or cumulative benefits based upon variable levels of supporting evidence [2,3].

Clinical practice guidelines issued by major oncology organizations, such as the European Society for Medical Oncology (ESMO), the National Comprehensive Cancer Network (NCCN), and the American Society of Clinical Oncology (ASCO), are one of the pillars for the translation of trial evidence into clinical practice recommendations. These guidelines differ in their methodology, including criteria for assessing the quality of evidence, defining recommendation strength, and dependence on expert consensus. As a result, recommendations for the same therapy may differ across guideline frameworks, particularly in situations where the evidence is still evolving or where outcomes vary across patient subgroups [4,5,6].

To complement guideline-based decision-making, ESMO introduced the Magnitude of Clinical Benefit Scale (MCBS) in 2015. Since its commencement, the scale has undergone a number of changes, with a recent update released in 2025 [7,8]. The scale evaluates survival endpoints, treatment toxicity, and QoL outcomes, with greater weight given to therapies that offer curative potential. Importantly, the scale applies distinct grading systems for curative (grades A–B) and palliative (scores 1–5) settings, recognizing the different therapeutic goals across disease contexts.

While ESMO-MCBS has emerged as a common instrument in clinical and scholarly literature [9,10], its relationship with treatment guideline recommendations across different oncology frameworks remains poorly described. While guidelines prioritize the level of evidence certainty and expert consensus, the MCBS gives precedence to the magnitude of clinical benefit. Notably, the degree to which these perspectives support each other in practice is not well understood.

Furthermore, systematic analyses of concordances of guideline systems for curative and palliative BC settings have not been performed. Given the never-ending stream of new agents in BC, coupled with the variable pricing/benefit profiles of those agents, a thorough knowledge of how value frameworks fit into guideline decisions is of the utmost importance. Identifying areas of concordance and disagreement across these systems may help to shed light on gaps in the definition and operationalization of value and is likely to impact future stewardship efforts.

Therefore, this study aims to evaluate the association between ESMO-MCBS scores and treatment guideline recommendations across ESMO, NCCN, and ASCO and to assess the concordance among these guideline frameworks in both curative and metastatic BC settings.

## 2. Methods

### 2.1. Study Design and Included Studies

This study used a comparative review methodology, based on published data, to explore associations between the ESMO-MCBS scores, the level of evidence (LOE), and the strength of recommendations (SOR) in major BC guidelines.

The analysis contained a dataset of 47 medications for BC whose ESMO-MCBS scores have been published on the official ESMO website (accessed on 30 June 2025) [11]. The ESMO-MCBS applies five evaluation forms based on the pivotal trial endpoint (OS, progression-free survival (PFS), or response rate), with adjustments for QoL and toxicity. Grades A–B reflect the highest benefit in curative settings, while scores 4–5 indicate substantial benefit in the palliative context [8].

### 2.2. Data Extraction and Categorization

A standardized data collection framework was applied. Variables included:

- Drug and trial information, such as tested agent, comparator/control, trial name, setting (curative/palliative), and BC subtype/subgroup.
- Guideline recommendation status from major oncology guidelines, including:

- ESMO Clinical Practice Guidelines [12], which provide recommendations graded according to strength of evidence (I–IV) and strength of recommendation (A–E), are often further expressed as strong, moderate, or weak recommendations (Table 1). Additionally, the ESMO-MCBS score will be reported [11].

- NCCN Guidelines [13] recommendations were classified according to their standardized categories of evidence and consensus (Categories 1, 2A, 2B, and 3), as defined in the NCCN framework (Table 2). In addition, treatment preference designations (e.g., “preferred,” “other recommended,” and “useful in certain circumstances”) were reported.

- ASCO Guidelines [14], which apply the GRADE framework [15] (Table 3). All guideline data were obtained from the most recent published versions of ESMO, NCCN, and ASCO BC guidelines.

### 2.3. Harmonization of Guideline Recommendation Categories

Guideline recommendations were subsequently recategorized into harmonized ordinal tiers to allow cross-framework comparison and correlation analyses. The categorization was primarily anchored in the level of evidence underlying each framework, aiming to preserve ordinal ranking rather than assume equivalence between categories. It should be noted that top-tier categories across frameworks are conceptually aligned and driven by high-quality evidence, and no recommendation categories are structurally comparable across all frameworks. However, the intermediate tier remains inherently heterogeneous, reflecting differing combinations of evidence quality, recommendation strength, and expert consensus across systems. Accordingly, analyses focused on rank-based relationships rather than assuming exact matching between categories.

For NCCN, recommendations were grouped into three ordered categories:

(1) *Category* 1 recommendations (including both “preferred” and “useful in certain circumstances”), reflecting the highest level of evidence and uniform expert consensus.
(2) *All other NCCN recommendation categories* (including Category 2A, 2B, and Category 3), representing lower levels of evidence or reduced consensus.
(3) *No NCCN recommendation* where a therapy was not addressed or endorsed in the guideline.

In addition, a sensitivity analysis was performed by restricting the highest recommendation tier to NCCN Category 1 ‘Preferred’ recommendations, excluding those classified as ‘useful in certain circumstances’.

For ASCO, recommendations were similarly harmonized into three ordinal tiers based on the strength of recommendation and the quality of evidence:

(1) *High*-*quality evidence with strong recommendation*.
(2) *All other ASCO recommendation categories*, including intermediate or low-quality evidence and/or conditional or weak recommendations.
(3) *No ASCO recommendation*, when no formal recommendation was issued due to insufficient evidence.

For ESMO, recommendations were recategorized as:

(1) *ESMO I*, *A* representing the strongest level of evidence and recommendation;
(2) *All other ESMO recommendation categories* (including I, B; I, C; II, A–C; and III);
(3) *No ESMO recommendation*.

**Figure 1:**
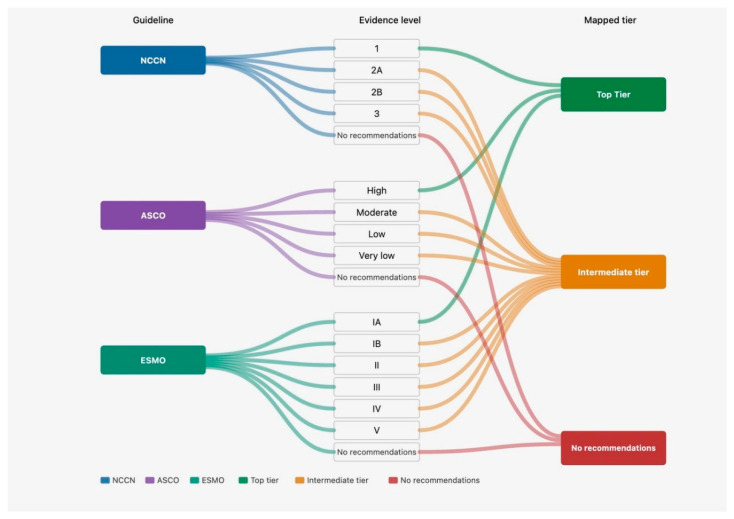
Conceptual mapping of guideline recommendation categories across NCCN, ASCO, and ESMO frameworks.

To enhance transparency and interpretability of this approach, a Sankey diagram (Figure 1) was developed to visually illustrate the mapping of original guideline categories into the harmonized tiers.

Sankey diagram illustrating the harmonization of guideline recommendation categories into three ordinal tiers (top, intermediate, and no recommendation). For NCCN, Category 1 recommendations (including “preferred” and “useful in certain circumstances”) were mapped to the top tier, while Categories 2A, 2B, and 3 were grouped into the intermediate tier. For ASCO, recommendations based on high-quality evidence with a strong strength of recommendation were classified as top tier, with all other recommendation categories grouped as intermediate. For ESMO, grade I, A recommendations were mapped to the top tier, with all other grades (I, B; I, C; II–V) categorized as intermediate. Therapies not addressed or not recommended in each guideline were classified as “no recommendation.” Flow widths are illustrative and represent conceptual mapping rather than quantitative weighting.

### 2.4. Statistical Analysis

Descriptive statistics were used to summarize categorical variables as frequencies and proportions. Analyses were conducted separately for palliative and curative settings.

Cross-tabulation analyses with Fisher’s exact test and Spearman’s rank correlation coefficient were used to evaluate the association between ESMO-MCBS scores and guideline recommendation categories, as both variables are ordinal and do not assume linearity or equal spacing between categories. Similarly, inter-guideline concordance was explored using cross-tabulation analyses with Fisher’s exact test to evaluate associations between recommendation categories and Spearman’s rank correlation to assess ordinal relationships between guideline systems.

In the curative setting, formal correlation and association testing were not performed due to the limited number of therapies and restricted variability in MCBS grades; results for this subgroup are therefore presented descriptively. All analyses were performed using Stata version 19.0 (StataCorp, College Station, TX, USA).

### 2.5. Ethical Considerations

As this project involves the secondary analysis of publicly available, published data with no direct patient involvement, no ethical approval is required.

## 3. Results

### 3.1. Overview of the Included Therapies

A total of 47 systemic therapies for BC were identified from the ESMO-MCBS website and included in the analysis. Thirty-eight (81%) agents were evaluated in the palliative setting, while 9/47 (19%) were evaluated in the curative/early BC setting (Table 4 and Table 5). Among the 38 therapies evaluated in the palliative setting, only 2 (5%) received the maximum ESMO-MCBS score of 5, with 34% scoring 4, 26% scoring 3, and 29% scoring 2. The lowest score of 1 was assigned to 2 therapies (5%). In contrast, among the nine therapies evaluated in the curative setting, the majority (6/9; 67%) received an ESMO-MCBS grade A, indicating substantial curative benefit. Two therapies (22%) were graded C, and one therapy (11%) had no assigned MCBS grade (missing classification).

Among the 47 therapies included, 27 (57%) were evaluated as part of a combination regimen, while the remaining 20 (43%) were tested as monotherapy. Regarding combination partners, endocrine therapies were the most frequent, used in 11 of 27 combinations (41%), most often with CDK4/6 inhibitors and PI3K/AKT pathway inhibitors. Chemotherapy backbones accounted for 10 of 27 combinations (37%). A smaller proportion (6 of 27 combinations; 22%) included other targeted agents, such as HER2-directed monoclonal antibodies, antibody–drug conjugates, or multi-agent targeted combinations.

Regarding the BC subtype, the largest subgroup consisted of HR-positive, HER2-negative disease, accounting for approximately 15 therapies (32%). HER2-positive BC represented the second most common category with 14 therapies (30%). A smaller subset targeted TNBC (5 therapies) and gBRCA-mutated HER2-negative disease (3 therapies). Several trials also focused on more granular biological groups such as HER2-low, PIK3CA-mutated, and ESR-1-mutated BC.

### 3.2. Trial Endpoints

Across the included trials, progression-free survival (PFS) was the most frequently used primary endpoint, appearing in approximately 55% of trials, while OS and PFS as co-primary endpoints were used in around 15%. Endpoints specific to early BC, such as disease-free survival (DFS/iDFS), accounted for approximately 15% of trials. A smaller number of studies used response-based endpoints, including objective response rate (ORR), pathological complete response (pCR), or time-to-progression. Secondary endpoints commonly included a combination of OS, PFS, ORR, quality-of-life measures, and safety outcomes. As secondary endpoints, OS was reported in 36 of 47 trials (77%), PFS in 17 trials (36%), and ORR in 30 trials (64%). Safety outcomes were reported in all the pivotal studies.

### 3.3. Approval Status

Among the evaluated therapies in this dataset, most agents (43/47, 92%) received FDA approval between November 2006 and January 2025. Only a limited subset does not hold FDA approvals for their tested indications. Specifically, bevacizumab, atezolizumab, and lapatinib/trastuzumab adjuvant combination are not FDA-approved therapies.

Among the 25 unique therapies, 7 (28%) received accelerated FDA approval. The following are the therapies and the tested setting for which accelerated approval was granted:

- Atezolizumab for PD-L1 ≥ 1% metastatic TNBC, based on IMpassion130.
- Bevacizumab for metastatic BC in combination with paclitaxel, based on E2100.
- Pertuzumab HER2-positive early BC in the neoadjuvant setting, supported by NeoSphere.
- Palbociclib received accelerated approval twice for HR+/HER2− metastatic BC in combination with letrozole, based on PALOMA-1/TRIO-18 and PALOMA-2.
- Pembrolizumab for PD-L1 CPS ≥ 10% metastatic TNBC, based on KEYNOTE-355.
- Sacituzumab govitecan received accelerated approval for pretreated metastatic TNBC, supported by the IMMU-132-01 single-arm study and later confirmed in ASCENT.
- Trastuzumab deruxtecan for heavily pretreated HER2-positive metastatic BC, supported by the DESTINY-Breast01 single-arm study and reinforced by DESTINY-Breast03.

All except for bevacizumab were subsequently converted to regular approval. However, the regular approval for atezolizumab in metastatic TNBC was later voluntarily withdrawn following failure to confirm clinical benefit. For approved therapies, the median lag between accelerated and regular approval was approximately 2.15 years (IQR 0.96–2.37 years). EMA approval was granted for 42 therapies (89%). The median time from regular FDA approval to EMA approval was 10.8 months (IQR 4.1–16.1 months). In several HER2-directed agents, EMA authorization preceded FDA approval.

### 3.4. Association Between Esmo-Mcbs and Guideline Recommendations

In the palliative setting, 15 therapies (40%) achieved high clinical benefit (ESMO-MCBS scores 4–5), while the remaining 23 therapies (60%) received scores of 1–3. Associations between ESMO-MCBS scores and guideline recommendations are summarized in Table 6.

Therapies with NCCN Category 1 recommendations were significantly more likely to demonstrate substantial clinical benefit compared with therapies in other NCCN categories or those without NCCN endorsement. Specifically, 62% of NCCN Category 1 therapies achieved MCBS scores of 4–5, compared with 13% of therapies in other NCCN categories, while none of the therapies without NCCN recommendations reached high MCBS scores (*p* = 0.003).

In contrast, no statistically significant association was observed between ASCO recommendation status and high MCBS scores. Among therapies supported by high-quality evidence and strong ASCO recommendations, 54% achieved MCBS scores of 4–5, compared with 42% among therapies receiving other ASCO recommendations; none of the therapies without ASCO recommendations demonstrated high MCBS scores (*p* = 0.101).

**Figure 2:**
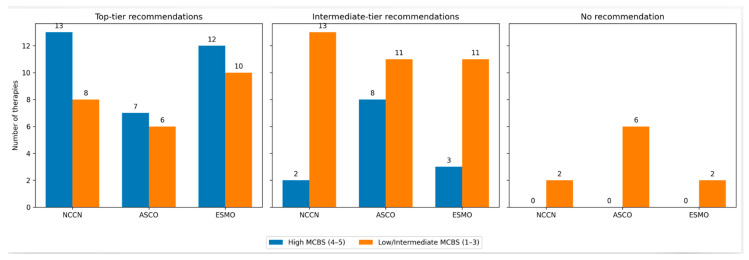
Association between guideline recommendation tiers and ESMO-MCBS scores across NCCN, ASCO, and ESMO frameworks. Top-tier recommendations refer to NCCN category 1 recommendations, ASCO high-quality evidence with strong recommendations, and ESMO grade I, A. ASCO: American Society of Clinical Oncology; ESMO-MCBS: European Society of Medical Oncology-Magnitude of Clinical Benefit Scale; NCCN: National Comprehensive Cancer Network.

Similarly, ESMO guideline recommendations were not significantly associated with MCBS scores. Among therapies receiving top-tier ESMO recommendation (I, A), 48% achieved MCBS scores of 4–5, compared with 27% among therapies assigned to other ESMO categories, while no therapies without ESMO recommendations demonstrated high clinical benefit (*p* = 0.073). The distribution of therapies across guideline recommendation tiers and ESMO-MCBS categories is summarized in Figure 2.

Spearman rank correlation analysis demonstrated a moderate positive correlation between palliative ESMO-MCBS scores and NCCN recommendation category (ρ = 0.48), indicating that therapies with a higher magnitude of clinical benefit were more likely to receive higher-tier NCCN recommendations. However, considerable variability remained, indicating that the association is not fully explanatory. In contrast, correlations between MCBS scores and ASCO (ρ = 0.18) and ESMO (ρ = 0.19) recommendation categories were weak.

In the curative setting, formal correlation and association analyses were not performed. This is due to the limited number of evaluated therapies (*n* = 9), the restricted range of ESMO-MCBS grades (A and C only), and the high degree of clustering within top-tier guideline recommendation categories, which together substantially limit statistical power and discriminative capacity (Table 7).

### 3.5. Inter-Guideline Associations and Rank Correlation Analysis

In the palliative setting, NCCN recommendations demonstrated a statistically significant association with ASCO recommendations, but not with ESMO classifications. Among therapies with NCCN Category 1 recommendations, 38% were supported by high-evidence, strong ASCO recommendations, compared with 33% among therapies with non–Category 1 NCCN recommendations. In contrast, therapies without NCCN recommendations uniformly lacked ASCO endorsement (*p* = 0.046). However, when the analysis was restricted to NCCN Category 1 “Preferred” therapies only, the association with high-evidence, strong ASCO recommendations was attenuated and no longer statistically significant, with 35% of preferred therapies receiving high-evidence ASCO endorsement compared with 37% among other NCCN-recommended therapies (*p* = 0.083).

Similarly, 15 of 21 (71%) therapies with NCCN Category 1 recommendations received ESMO I, A endorsement. By comparison, only 7 of 15 (47%) therapies with non–Category 1 NCCN recommendations were classified as ESMO I, A, while none of the therapies without NCCN recommendations achieved top-tier ESMO endorsement. However, these associations did not reach statistical significance (*p* = 0.075). Restricting the analysis to NCCN Category 1 “Preferred” therapies did not strengthen the association (*p* = 0.266). These results are presented in Table 8 and Table 9.

**Figure 3:**
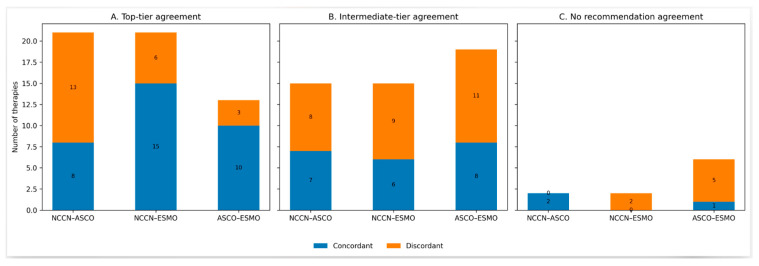
Concordance of guideline recommendations across harmonized tiers in the palliative setting. Top-tier recommendations refer to NCCN category 1 recommendations, ASCO high-quality evidence with strong recommendations, and ESMO grade I, A. ASCO: American Society of Clinical Oncology; ESMO-MCBS: European Society of Medical Oncology-Magnitude of Clinical Benefit Scale; NCCN: National Comprehensive Cancer Network.

The strongest association was observed between ASCO and ESMO classifications. Therapies supported by high-evidence, strong ASCO recommendations were predominantly classified as ESMO I, A (10 of 13, 77%), whereas therapies in lower ASCO recommendation categories showed greater dispersion across ESMO tiers. Therapies without ASCO recommendations mostly fell within intermediate ESMO categories. This association was statistically significant (*p* = 0.033) (Table 10). Figure 3 illustrates the concordance and discordance between guideline recommendations across harmonized recommendation tiers.

Rank correlations among guideline systems ranged from weak to moderate. The strongest Spearman correlation was observed between ASCO and ESMO (ρ = 0.48), followed by NCCN and ESMO (ρ = 0.41), whereas the correlation between NCCN and ASCO was weaker (ρ = 0.28).

## 4. Discussion

This study describes a structured comparison between the concordance of ESMO-MCBS scores and treatment recommendations given by ESMO, NCCN, and ASCO in both curative and palliative BC settings. By aligning recommendation tiers under the different frameworks and restricting analyses of concordance and discordance to officially published gradings, the current study gives a transparent evaluation of interframework variability. Overall, there were substantial discrepancies in the positioning of therapy, with the largest discrepancies seen in the metastatic setting.

The therapeutic portfolio of included agents reflects contemporary BC drug development. Most therapies were evaluated in the palliative setting and frequently tested as combination regimens in biologically defined subgroups. ESMO-MCBS scores in metastatic disease were broadly distributed across intermediate tiers, whereas the majority of curative therapies achieved grade A. Selection of endpoints differed substantially between settings: PFS predominated in metastatic trials, while DFS/iDFS was most common in early BC. This distinction is critical, as endpoint choice shapes evidence maturity, regulatory approval pathways, and subsequent recommendation strength. Notably, 28% of therapies initially received accelerated FDA approval, often based on surrogate endpoints or early efficacy signals in high-unmet-need populations [16]. This highlights how emerging evidence can support the early adoption of potential therapies in clinical practice before mature data are available, particularly in advanced disease.

### 4.1. Association Between Esmo-Mcbs and Guideline Recommendations

In the palliative setting, the association between ESMO-MCBS scores and guideline recommendations was present but incomplete. While 40% of therapies achieved high clinical benefit (scores 4–5), the majority (60%) fell within intermediate or low magnitude tiers (scores 1–3), creating a heterogeneous evidentiary landscape. A significant association was observed between ESMO-MCBS and NCCN recommendations: therapies receiving NCCN Category 1 endorsement were substantially more likely to demonstrate high clinical benefit, and none of the therapies without an NCCN recommendation achieved high ESMO-MCBS scores. In contrast, the association with ASCO recommendations did not reach statistical significance, as high-evidence, strong ASCO recommendations were distributed across both high and intermediate ESMO-MCBS tiers (54% vs. 42%). Thus, although categorical overlap existed, the overall level of concordance across frameworks remained modest.

Substantial discordance was also observed between ESMO guideline recommendations and ESMO-MCBS scores. Among 22 therapies receiving the highest ESMO recommendation (I, A), nearly half were associated with low ESMO-MCBS scores (1–3), including palbociclib (PALOMA-2), abemaciclib (MONARCH 2/3), trastuzumab emtansine (EMILIA), talazoparib (EMBRACA), eribulin (EMBRACE), everolimus (BOLERO-2), trastuzumab deruxtecan (early context; DESTINY-Breast06), and PI3K/AKT-pathway inhibitors such as inavolisib and capivasertib. This apparent paradox reflects the distinct constructs underlying each system. ESMO guidelines are developed using the GRADE methodology, which evaluates certainty of evidence and strength of recommendation across domains such as risk of bias, consistency, indirectness, imprecision, and balance of benefits and harms [15]. High-certainty evidence may therefore justify strong recommendations even when absolute survival gains are modest. In contrast, the ESMO-MCBS applies predefined absolute thresholds to quantify magnitude of benefit [8]. Strength of recommendation and magnitude of benefit are thus related but non-equivalent dimensions of value assessment.

This difference is not unique to the ESMO framework but reflects a broader distinction between value assessment tools and clinical practice guidelines. Guideline recommendations from NCCN, ASCO, and ESMO are largely driven by the strength and certainty of evidence, together with clinical context and expert consensus. In contrast, the ESMO-MCBS focuses on quantifying the magnitude of clinical benefit using predefined thresholds. While elements of evidence strength are indirectly considered within its scoring system, its primary aim is to measure how much benefit a treatment provides rather than how confidently it is supported. As a result, these approaches address related but different aspects of therapeutic value, which may contribute to the observed differences between them. This distinction helps explain the variability in concordance observed across frameworks.

Within this context, the 4th ESO–ESMO International Consensus Guidelines for Advanced Breast Cancer (ABC 4) acknowledged the role of the ESMO-MCBS as a complementary tool to quantify clinical benefit. Within this framework, therapies may receive strong consensus recommendations based on evidence quality and clinical need, even when the magnitude of benefit—as reflected by ESMO-MCBS—is modest. By providing standardized and explicit quantification, ESMO-MCBS enables clinicians, policymakers, and other stakeholders to better contextualize recommendations, support resource prioritization, and enhance confidence in decision-making [17].

### 4.2. Broader Oncology Context and Systematic Nature of Discordance

The discordance seen in this cohort is similar to patterns seen in the larger oncology approval landscape. Analyses of EMA-approved cancer drugs have shown that at the time of approval, only 35% of indications demonstrated OS benefit and 10% demonstrated QoL improvement; even after extended follow-up, 49% remained of uncertain clinical value, and fewer than half met ESMO-MCBS thresholds for clinically meaningful benefit [3]. This regulatory context is especially pertinent to the setting of metastatic BC, in which surrogate endpoints and accelerated approvals are commonplace [18].

Most importantly, this study captured the fact that discordance was systematic rather than random. Therapies driving divergence shared common characteristics of relying on PFS as the primary endpoints, modest or evolving OS benefit, biomarker-selected populations, a significant toxicity burden, and frequent use in heavily pretreated or post-CDK4/6 settings. These agents were appropriately endorsed by guideline frameworks based on robust randomized evidence and clinical need [3,18,19], yet did not meet ESMO-MCBS thresholds for high magnitude of benefit because absolute survival gains were limited or immature [2,7].

By contrast, descriptive alignment seemed to be stronger in the curative setting. Most early-stage therapies achieved ESMO-MCBS grade A and received top-tier guideline endorsement, due to more stable time-to-event endpoints (DFS/iDFS, OS), clearer therapeutic intent, and more durable benefit estimation. Metastatic trials, in contrast, are inherently more vulnerable to surrogate reliance, post-progression inferences, and changing standards of care. This may make it difficult to determine long-term survival impact and exaggerate the divergence in evaluative systems [3,20].

Changes in the ESMO-MCBS framework over time may also contribute to apparent discordance. For example, the ESMO-MCBS scores assigned to agents such as trastuzumab emtansine, palbociclib, abemaciclib, alpelisib, and everolimus have been revised and updated across successive versions of the scale. These updates reflect refinements in scoring thresholds as longer-term efficacy and toxicity data emerge and are an inherent feature of clinical value assessment [11].

### 4.3. Inter-Guideline Association

Beyond comparisons with ESMO-MCBS, meaningful variability was observed across NCCN, ASCO, and ESMO recommendations, particularly in the palliative setting. This pattern is consistent with previous analyses showing increasing divergence as therapeutic aims move away from cure towards disease control, where reliance on surrogate endpoints, smaller effect sizes, and more heterogeneous populations allows greater interpretative flexibility [21].

Differences in guideline revision cycles likely contribute to this variability. NCCN operates as a continuously updated (“living”) guideline, incorporating new evidence and regulatory approvals through interim revisions. This structure enables relatively rapid endorsement of therapies supported by emerging phase III or accelerated approval data [13]. Agents such as sacituzumab govitecan, trastuzumab deruxtecan, inavolisib, and capivasertib entered NCCN recommendations while survival data remained immature. In contrast, ASCO and ESMO updates generally go through more formal review processes that will delay top-tier endorsement until evidence maturity is judged to be sufficient [14,15].

Methodological orientation also plays a role. While NCCN integrates expert consensus and real-world feasibility more prominently, both ASCO and ESMO rely on structured evidence appraisal processes when assigning recommendation strength [21]. This likely explains why the strongest association was observed between ASCO and ESMO (*p* = 0.033). Concordance was greatest for therapies supported by mature OS data, whereas variation was concentrated among intermediate-benefit, surrogate-driven therapies, which predominate in metastatic BC.

These discrepancies do not reflect conflicting clinical goals. Instead, they arise from variations in update frequency, evidentiary standards, and the balance between expert consensus and methodological rigor. In the metastatic setting, where evidence evolves rapidly, and absolute survival gains are often modest, these structural differences frequently lead to divergent recommendations across frameworks [13,21].

### 4.4. Practical Implications

Clinicians ideally seek consistency and confidence in guidance to limit unwarranted variation and facilitate evidence-based care. However, some degree of discrepancy may be informative. Broader endorsement within one framework may facilitate earlier access in certain health systems, while more conservative frameworks signal where benefit magnitude is modest and prioritization may be warranted [22]. In the case of metastatic cancer, where therapeutic sequencing and resource allocation are so crucial, the ESMO-MCBS provides a complementary layer to stratify the magnitude of benefit, alongside consensus-driven guidance designation. This distinction is particularly relevant for reimbursement and formulary decision-making, where separating “endorsement” from “magnitude of benefit” may support more transparent prioritization and value-based resource allocation [22].

Our findings support the potential value of an integrated cross-guideline approach that distinguishes between therapies that are guideline-recommended and those that provide substantial clinical benefit. Such differentiation would be particularly useful for pharmacy and therapeutics committees evaluating intermediate-benefit therapies, where discordance was most concentrated.

## 5. Strengths and Limitations

A major strength of this study is its exclusive reliance on officially published guideline recommendations and ESMO-MCBS scores, avoiding subjective reinterpretation or recalculation of clinical benefit. This approach enhances methodological rigor, minimizes investigator-related bias, and improves reproducibility. Additionally, harmonizing recommendation categories into ordinal tiers enabled cross-framework comparison while preserving the relative ranking of recommendations across systems.

Several limitations should be acknowledged. The dataset was limited to therapies with available ESMO-MCBS scores, which may affect the representativeness of the broader treatment landscape; however, this was a deliberate methodological choice to ensure standardized and reproducible evaluation. The number of therapies, particularly in the curative setting, was limited, restricting the ability to perform formal statistical analyses in this subgroup. Additionally, guideline recommendations represent time-specific snapshots and may evolve as new evidence emerges.

Furthermore, while harmonization was necessary for cross-framework comparison, it simplifies the complexity of individual guideline systems and may introduce misclassification, particularly within intermediate categories that are not strictly homogeneous across frameworks. To address this, analyses were based on ordinal relationships and supported by sensitivity analyses. This approach is consistent with prior comparative studies that emphasize relative ranking rather than strict equivalence between categories [21].

Finally, given the relatively small sample size and multiple comparisons performed without formal adjustment, findings should be interpreted with caution, as some statistically significant associations may reflect chance (type I error). As the analyses were exploratory, adjustment for multiplicity was not applied to avoid excessive type II error, and emphasis was placed on the overall consistency and direction of findings.

## 6. Conclusions

In BC, a significant association exists across ESMO, NCCN, and ASCO for therapies supported by mature, high-certainty evidence. However, meaningful variability persists—particularly in the metastatic setting—where surrogate endpoints, accelerated approvals, and modest absolute survival gains predominate. Discordance reflects structural and philosophical differences in how evidence is graded and benefit is quantified rather than inconsistency in clinical intent.

The ESMO-MCBS appears most valuable as a complementary instrument, adding standardized magnitude-of-benefit assessment to consensus-driven guideline endorsement. Greater transparency regarding both recommendation strength and absolute benefit may enhance shared decision-making, support rational reimbursement policies, and improve stewardship in advanced disease. Future research should examine longitudinal changes in guideline alignment and explore structured integration of value frameworks into multidisciplinary decision-making processes.

## Figures and Tables

**Table 1:** European Society of Medical Oncology (ESMO) level of evidence and grade of recommendation classification [12].

Level of Evidence
I Evidence from at least one large randomized, controlled trial of good methodological quality (low potential for bias) or meta-analyses of well-conducted randomized trials without heterogeneity II Small randomized trials or large randomized trials with suspicion of bias (lower methodological quality) or meta-analyses of such trials or of trials with demonstrated heterogeneity III Prospective cohort studies IV Retrospective cohort studies or case–control studies V Studies without control group, case reports, expert opinions
Grades of Recommendation
A Strong evidence for efficacy with a substantial clinical benefit, strongly recommended B Strong or moderate evidence for efficacy but with limited clinical benefit, is generally recommended C Insufficient evidence for efficacy or benefit does not outweigh the risk or the disadvantages (adverse events, costs, etc.), optional D Moderate evidence against efficacy or for adverse outcome, generally not recommended E Strong evidence against efficacy or for adverse outcome, never recommended

**Table 2:** National Comprehensive Cancer Network (NCCN) guideline categories of evidence, consensus, and treatment preference ratings [13].

NCCN Ratings	NCCN Definitions
Level of Evidence and Consensus	
1	High level evidence with uniform panel consensus (>85% of votes)
2A	Lower level evidence with uniform panel consensus (>85% of votes)
2B	Lower level evidence without uniform consensus (50–85% of votes)
3	Any level evidence but with major disagreement (25–50% of votes)
Treatment Preference	
Preferred	Based on superior efficacy, safety, and evidence and, when appropriate, affordability
Alternative preferred	No definition given
Other recommended	May be somewhat less efficacious, more toxic, or based on less mature data; or significantly less affordable for similar outcomes
Useful in certain circumstances	May be used for select patient populations defined with recommendation

**Table 3:** Levels of evidence quality and definitions according to the American Society of Clinical Oncology (ASCO) framework [14].

Grade	Definition
High	We are very confident that the true effect lies close to that of the estimate of the effect
Moderate	We are moderately confident in the effect estimate: The true effect is likely to be close to the estimate of the effect, but there is a possibility that it is substantially different
Low	Our confidence in the effect estimate is limited: The true effect may be substantially different from the estimate of the effect
Very Low	We have very little confidence in the effect estimate: The true effect is likely to be substantially different from the estimate of effect

**Table 4:** Therapies evaluated in the palliative setting.

Tested Agent	Trial Name (Phase)	Subtype	Combined Agent (If Applicable)	Control Arm	ESMO-MCBS Grade
Abemaciclib	MONARCH 3 (III)	HR+, HER2−	Aromatase inhibitor	Placebo + aromatase inhibitor	2
Abemaciclib	MONARCH 2 (III)	HR+, HER2−	Fulvestrant	Fulvestrant + placebo	4
Abemaciclib	MONARCH 1 (II)	HR+, HER2−	NA	Single arm	3
Alpelisib	SOLAR-1 (III)	HR+, HER2−, PIK3CA-mut	Fulvestrant	Placebo + fulvestrant	2
Atezolizumab	IMpassion130 (III)	TNBC, PD-L1 ≥ 1%	Nab-paclitaxel	Placebo + nab-paclitaxel	3
Bevacizumab	RIBBON-1 (III)	NA	Capecitabine	Placebo + capecitabine	3
Bevacizumab	E2100 (III)	NA	Paclitaxel	Paclitaxel	2
Capivasertib	CAPItello-291 (III)	PIK3CA, AKT1, PTEN−, HR+, HER2−	Fulvestrant	Placebo + fulvestrant	3
Dato-DxD	TROPION-Breast01 (III)	HR+, HER2−	NA	Investigator’s choice of chemotherapy (eribulin, capecitabine, vinorelbine, or gemcitabine)	3
Elacestrant	EMERALD (III)	ER+, HER2−, ESR1-mut	NA	Standard-of-care endocrine therapy	3
Eribulin	Study 305 and Study 301 (Pooled analysis) (III)	HER2−	NA	Treatment of physician’s choice	1
Eribulin	EMBRACE (III)	NA	NA	Treatment of physician’s choice	2
Everolimus	BOLERO-2 (III)	HR+, HER2−	Exemestane	Exemestane + placebo	2
Fulvestrant	FALCON (III)	ER+	NA	Anastrozole	2
Inavolisib	INAVO120 (III)	HR+, HER2−, PIK3CA-mut	Palbociclib + fulvestrant	Placebo + palbociclib + fulvestrant	3
Lapatinib	EGF104900 (III)	HER2+, HR−	Trastuzumab	Lapatinib	4
Lapatinib	EGF100151 (III)	HER2+	Capecitabine	Capecitabine	2
Margetuximab	SOPHIA (III)	HER2+	Chemotherapy	Trastuzumab + chemotherapy	2
Neratinib	NALA (III)	HER2+	Capecitabine	Lapatinib + capecitabine	1
Olaparib	OlympiAD (III)	HER2−, gBRCA1/2-mut	NA	Standard chemotherapy	4
Palbociclib	PALOMA-3 (III)	HR+, HER2−	Fulvestrant	Fulvestrant + placebo	4
Palbociclib	PALOMA-2 (III)	HR+, HER2−	Letrozole	Letrozole + placebo	2
Palbociclib	PALOMA-1/TRIO-18 (II)	HR+, HER2−	Letrozole	Letrozole	3
Pembrolizumab	KEYNOTE-355 (III)	TNBC, PD-L1 CPS ≥ 10%	Chemotherapy	Placebo + chemotherapy	4
Pertuzumab	CLEOPATRA (III)	HER2+	Trastuzumab + docetaxel	Trastuzumab + docetaxel + placebo	4
Ribociclib	MONALEESA-7 (III)	HR+, HER2−	Endocrine therapy	Placebo + endocrine therapy	5
Ribociclib	MONALEESA-3 (III)	HR+, HER2−	Fulvestrant	Placebo + fulvestrant	4
Ribociclib	MONALEESA-2 (III)	HR+, HER2−	Letrozole	Letrozole + placebo	4
SG	TROPiCS-02 (III)	HR+, HER2−	NA	Physician’s choice chemotherapy (eribulin, vinorelbine, capecitabine, or gemcitabine)	4
SG	IMMU-132-01 (I/II)	TNBC	NA	Single arm	2
SG	ASCENT (III)	TNBC	NA	Physician’s choice of single agent ChT	5
Talazoparib	EMBRACA (III)	HER2−, gBRCA1/2-mut	NA	Standard ChT	3
TDXd	DESTINY-Breast06 (III)	HER2-low, HR+, HER2-ultralow	NA	Investigator’s choice ChT (capecitabine, paclitaxel, nab-paclitaxel)	3
TDXd	DESTINY-Breast04 (III)	HER2-low	NA	Pysician’s ChT choice (eribulin, capecitabine, gemcitabine, nab-paclitaxel, or paclitaxel)	4
TDXd	DESTINY-Breast03 (III)	HER2+	NA	T-DM1	4
TDXd	DESTINY-Breast01 (II)	HER2+	NA	Single arm	2
TDM-1	EMILIA (III)	HER2+	NA	Lapatinib + capecitabine	4
Tucatinib	HER2CLIMB (II/III)	HER2+	Trastuzumab + capecitabine	Placebo, trastuzumab + capecitabine	4

ChT: chemotherapy, Dato-DxD: Datopotamab deruxtecan, SG: Sacituzumab govitecan, TDXd: Trastuzumab deruxtecan, TDM-1: Trastuzumab emtansine, NA: Not applicable.

**Table 5:** Therapies evaluated in the curative setting.

Tested Agent	Trial Name (Phase)	Subtype	Combined Agent (If Applicable)	Control Arm	ESMO-MCBS Grade
Olaparib	OlympiA (III)	HER2−, gBRCA-mut	NA	Placebo	A
Pertuzumab	NeoSphere (II)	HER2+	Trastuzumab + docetaxel	Trastuzumab + docetaxel	C
Ribociclib	NATALEE (III)	HR+, HER2−	NASI	NSAI	A
Abemaciclib	monarchE (III)	HR+, HER2−	Standard endocrine therapy (aromatase inhibitors and/or antiestrogens ± ovarian suppression)	Standard endocrine therapy	A
Pembrolizumab	KEYNOTE-522 (III)	TNBC	ChT (paclitaxel + carboplatin, followed by doxorubicin–cyclophosphamide or epirubicin–cyclophosphamide)	Placebo + ChT (paclitaxel + carboplatin, followed by doxorubicin–cyclophosphamide or epirubicin–cyclophosphamide)	A
TDM-1	KATHERINE (III)	HER2+	NA	Trastuzumab	A
Trastuzumab (1 year)	HERA (III)	HER2+	NA	Observation	A
Neratinib	ExteNET (III)	HR+, HER2+	NA	Placebo	NA
Pertuzumab	APHINITY (III)	HER2+	Standard adjuvant ChT + 1 year of treatment with trastuzumab	Placebo + standard adjuvant ChT + 1 year of treatment with trastuzumab	C

ChT: chemotherapy, NASI: non-steroidal aromatase inhibitor, TDM-1: Trastuzumab emtansine, NA: Not applicable.

**Table 6:** Association between guideline recommendations and ESMO-MCBS scores.

Guideline	Recommendation Category	ESMO-MCBS Score		Total
		4,5	1–3	
**NCCN**	Category 1 (Preferred, Useful in certain circumstances)	13	8	21
	All other categories	2	13	15
	No recommendation	0	2	2
	Total	15	23	38
	Fisher’s exact *p*-value = 0.003			
**ASCO**	Evidence quality: high, strength of recommendation: strong	7	6	13
	All other categories	8	11	19
	No recommendation	0	6	6
	Total	15	23	38
	Fisher’s exact *p*-value = 0.101			
**ESMO**	I, A	12	10	22
	All other categories	3	11	14
	No recommendation	0	2	2
	Total	15	23	38
	Fisher’s exact *p*-value = 0.073			

ESMO-MCBS: European Society of Medical Oncology-Magnitude of Clinical Benefit Scale.

**Table 7:** ESMO-MCBS grades and guideline recommendations in the curative setting.

Tested Agent	ESMO-MCBS Grade	NCCN Recommendation	ASCO Recommendation	ESMO Recommendation
Olaparib	A	Category 2A + Preferred	moderate, conditional/moderate	I, A
Pertuzumab (Neoadjuvant)	C	Category 1 + Preferred	high, strong	I, A
Ribociclib	A	Category 1 + Preferred	high, conditional	NA
Abemaciclib	A	Category 1 + Preferred	high, strong	I, A
Pembrolizumab	A	Category 1 + Preferred	moderate, strong	I, A
TDM-1	A	Category 1 + Useful in certain circumstances	high, strong	I, A
Trastuzumab (1 year)	A	Category 1 + Preferred	high, strong	I, A
Neratinib	NA	Category 1 + Preferred	high, moderate	I, A
Pertuzumab (adjuvant 1 year)	C	Category 2A + Preferred	moderate, conditional/moderate	I, A

ASCO: American Society of Clinical Oncology, ESMO-MCBS: European Society of Medical Oncology-Magnitude of Clinical Benefit Scale, NCCN: National Comprehensive Cancer Network. NA: not available.

**Table 8:** Association between NCCN and ASCO guideline recommendations in the palliative setting.

NCCN Recommendation Category	ASCO Recommendation Category			Total
	High Evidence, Strong	All Other Categories	No Recommendation	
Category 1 (Preferred, Useful in certain circumstances)	8	12	1	21
All other categories	5	7	3	15
No recommendation	0	0	2	2
Total	13	19	6	38

Fisher’s exact *p*-value = 0.046; ASCO: American Society of Clinical Oncology, NCCN: National Comprehensive Cancer Network.

**Table 9:** Association between NCCN and ESMO guideline recommendations in the palliative setting.

NCCN Recommendation Category	ESMO Recommendation Category			Total
	I, A	All Other Categories	No Recommendation	
Category 1 (Preferred, Useful in certain circumstances)	15	6	0	21
All other categories	7	6	2	15
No recommendation	0	2	0	2
Total	25	11	2	38

Fisher’s exact *p*-value = 0.075. ESMO: European Society of Medical Oncology, NCCN: National Comprehensive Cancer Network.

**Table 10:** Association between ASCO and ESMO guideline recommendations in the palliative setting.

ASCO Recommendation Category	ESMO Recommendation Category			Total
	I, A	All Other Categories	No Recommendation	
Evidence quality: high, strength of recommendation: strong	10	2	1	13
All other categories	11	8	0	19
No recommendation	1	4	1	6
Total	25	11	2	38

Fisher’s exact *p*-value = 0.033; ASCO: American Society of Clinical Oncology, ESMO: European Society of Medical Oncology.

## Data Availability

The data used in this study were obtained from publicly available sources, including published clinical trials and guideline documents. All relevant data are included within the article. Further inquiries can be directed to the corresponding author.
